# Are Japanese Randomized Controlled Trials Up to the Task? A Systematic Review

**DOI:** 10.1371/journal.pone.0090127

**Published:** 2014-03-03

**Authors:** Daisuke Yoneoka, Akinori Hisashige, Erika Ota, Karin Miyamoto, Shuhei Nomura, Miwako Segawa, Stuart Gilmour, Kenji Shibuya

**Affiliations:** 1 Department of Global Health Policy, Graduate School of Medicine, University of Tokyo, 7-3-1 Hongo, Bunkyo-ku, Tokyo, Japan; 2 Institute of Healthcare Technology Assessment, 2-24-10, Shomachi, Tokushima, Japan; McGill University, Canada

## Abstract

**Objectives:**

Despite increasing numbers of RCTs done in Japan, existing international databases fail to capture them, and detailed information on the quality of Japanese RCTs is still missing. This study assessed the characteristics and quality of Japanese RCTs and analyzed factors related to their quality.

**Methods:**

All RCTs conducted in Japan, and published as original articles that assessed the effect of healthcare interventions on humans in 2010, were included. We excluded study protocols, conference abstracts, and comments. In addition, quasi-RCTs were excluded. Data were independently abstracted and assessed by two of the authors and disagreements were resolved by consensus. The quality of Japanese RCTs randomly sampled was assessed using the method guidelines for systematic reviews from the Cochrane Back Review Group. The factors affecting RCT quality were analyzed using a logistic regression model.

**Results:**

A total of 1013 RCTs conducted in Japan were published in 2010. The majority was small-scale (55% of RCTs with sample size less than 50). Eighty percent of RCTs had no information on the funding source and only 8% had been registered before their implementation. RCTs not indexed in international databases were a moderate number (118 RCTs: 37.7% of non-indexed RCTs were of high quality). Surgical intervention studies for external causes of morbidity and mortality with a large sample size, trial registration and a large number of arms were most likely to be of higher quality.

**Conclusion:**

Despite a considerable number of RCTs conducted in Japan, their quality is not satisfactory in some domains. On the other hand, there are high-quality, non-indexed RCTs. The full disclosure of trial information and quality control of clinical trials are urgently needed in Japan.

## Introduction

Randomized controlled trials (RCTs) are widely regarded as providing reliable evidence on the benefits of healthcare interventions. The number of published RCTs is rapidly increasing worldwide [1], but the validity of all RCTs depends on the underlying methodologies (e.g. allocation concealment, blinding, intention-to-treat analysis, and loss to follow-up) which are considered to be important to the quality of RCTs [2]. Previous evidences showed that poor-quality trials might lead to the biased estimation of the true effects of interventions [3]–[5].

A systematic review of trends in the methodological quality of RCTs showed there were improvement of quality in some methodological aspects over time, but others did not significantly improve [6]. Many methods for improving their quality have been proposed. For example, the CONSORT Statement was developed to provide the guideline to improve the quality of reporting [3], [7], and journal endorsement of CONSORT has been shown to be positively related with better quality of reporting of randomized trials [3], [7].

The number of clinical trials cited in 2007 and reported during the last 60 years in Japan was ranked 7^th^ and 9^th^ in the world, respectively. While RCTs conducted in Japan have been partly reported among international journals and databases, most have been paid little attention from an international perspective [1], [8]. For example, the majority of Cochrane systematic reviews did not search Japanese databases because of the language barrier [9], [10]. Therefore, even though Japan has made a relatively large contribution to the evaluation of healthcare interventions, comprehensive information and usefulness on RCTs conducted in Japan is still missing. The major objectives of the present study are therefore to systematically review RCTs conducted in Japan. We identified the number, general characteristics, and quality of all Japanese RCTs published in Japan in 2010, and analyzed factors associated with their quality.

## Methods

### Search strategy

RCTs conducted in Japan and published in 2010 were identified through two broad sources. Firstly, we searched the domestic Japanese database: the Japan Medical Abstract Society Database (January 1 to December 31, 2010). This database includes studies published in Japanese journals, which are written in Japanese sometimes with English summary. Secondly, we searched international databases: MEDLINE (January 1 to December 31, 2010), EMBASE (January 1 to December 31, 2010), CINAHL (January 1 to December 31, 2010) and PsycINFO (January 1 to December 31, 2010). These databases include Japanese papers written in English, as well as only English summaries for a very limited number of papers written in Japanese. We included search terms related to RCTs such as “single blind”, “double blind”, “placebo”, “randomized controlled trials”, “randomization” and “random* (wild card)”, and articles published from Japan (final search: November 11, 2011). The search terms are presented in Appendix S1. The search strategies were designed by the first author and a librarian and were appropriately modified for each database. This systematic review has no protocol for the study.

### Inclusion and exclusion criteria

All papers reporting RCTs conducted in Japan and published as original articles in 2010, which assessed a healthcare intervention, were included. The definition of RCTs followed Cochrane review guidelines. We considered RCTs that took place in Japan and for which the affiliation of the first author was located in Japan. We excluded study protocols, conference abstracts, and comments, as well as quasi-RCTs such as allocation by date of birth, day of the week or medical record number.

### Study selection and data extraction

Eligible studies were identified by one reviewer based on available information in the title, abstract, and/or full text. A second reviewer independently checked articles that were judged by the first reviewer to be suitable for inclusion. Discrepancies were resolved by discussion and consensus. The title and abstract were initially scanned for relevance. If it was difficult to determine the trial characteristics, full texts were obtained and checked. The data for the following characteristics of each trial were extracted in this way: type of intervention, disease and conditions, study design, type of control group, number of arms, sample size, registration, funding sources and publisher.

### Assessment of study quality

Sixty percent of all RCTs identified were randomly selected to evaluate their methodological quality. This sample size was determined on the basis that it would be sufficient to identify more than a 10% difference in proportions of high quality between indexed and non-indexed groups using a chi-squared test, given the available total number of selected RCTs [11]. Two independent reviewers assessed the quality of each trial using the 2009 updated method guidelines for systematic reviews from the Cochrane Back Review Group, which can be regarded as the detailed version of the risk of bias tool created by the Cochrane Systematic Reviews Group, and has confirmed internal/external validity and intra-rater/inter-rater reliability [12], [13]. The 2009 updated method guidelines for systematic reviews consist of 12 criteria for assessing the quality of an RCT based primarily on published reports. Disagreements were resolved by consensus, and a third reviewer was consulted if disagreements persisted. We did not attempt to contact the corresponding authors to assess the quality of the data and based our review only on reported information. RCTs for which blinding was impossible but outcome measurements were objective bio-marker based assessments (such as mortality) were scored “low risk of bias” according to Cochrane review guidelines. RCTs with subjective outcomes (such as quality of life scales) and no blinding were scored “high risk of bias” according to Cochrane review guidelines. This categorization is consistent with Cochrane review guidelines, which state that a study can be scored “low risk of bias” when there was “no blinding of outcome assessment, but the review authors judge that the outcome measurement is not likely to be influenced by lack of blinding.” [14].

### Data analysis

We first identified the number of RCTs conducted in Japan in 2010 and examined their characteristics. The quality of the randomly sampled Japanese RCTs was assessed using the 2009 updated method guideline for systematic reviews [12], [14]. To compare the quality of Japanese RCTs according to the type of journals, they were classified into three categories: those indexed in international databases and published in international journals, those indexed in international database with only English summary, and those not indexed in international databases and published in Japanese journals only. In statistical analysis, the chi-square test or Fisher's exact test was used with Holm's multiple corrections. Finally, the factors affecting RCT quality were analyzed using a logistic regression model. The dependent variable was a binary variable indicating whether the trial was high quality or not. The studies were rated as having a “low risk of bias” when at least six of the 12 items were scored as “yes”. Studies with less than six items having low risk of bias or with serious flaws were rated as having a “high risk of bias” following the method of Furlan et al. (2009) [12]. Under this method, a serious flaw is defined as a drop-out rate in one group of over 80% and a non-compliance rate of over 50%. The independent variables were disease and conditions, type of intervention, comparison group, study design, sample size, registration of the study protocol, number of arms, international indexing and publisher and description of funding sources. Because it was not possible to categorize sample size on the basis of its statistical adequacy for every paper, sample size was instead divided into several categories. Sample size was categorized based on observable breakpoints seen in the distribution of sample sizes, calculated using kernel density estimation. Statistical analyses were performed with R version 2.15.1 (Free Software Foundation's GNU General Public License). A p-value less than 0.05 was considered statistically significant.

## Results

**Figure 1:**
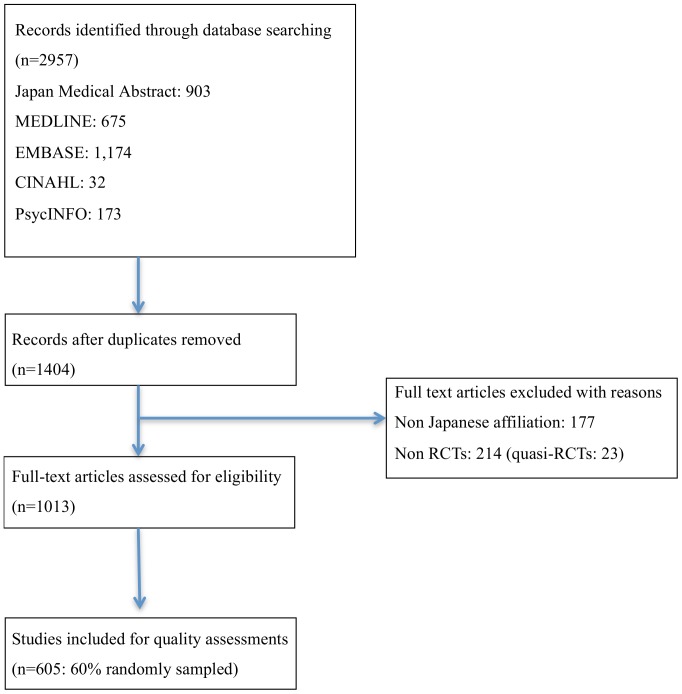
Study selection flow diagram.

Figure 1 shows the flow diagram describing the study identification strategy. The Japan Medical Abstract Society database, MEDLINE, EMBASE, CINAHL, and PsycINFO were searched, and 2957 articles were found (EMBASE: 1174; Japan Medical Abstract database: 903; MEDLINE: 675; PsycINFO: 173 and CINAHL: 32). 1013 articles met the inclusion criteria and 1944 articles were excluded because they were duplicates (79.9%), not RCTs (11.0%, of which 5.9% were quasi-RCTs) or had non-Japanese authorship (9.1%). The 1013 articles included consisted of 378 articles (37.3%) indexed in international databases and published in international journals, 105 articles (10.4%) indexed in international databases and published in Japanese journals and 530 articles (52.3%) non-indexed in international databases and published in Japanese journals.

The three most frequent diseases and conditions based on ICD-10 were diseases of the circulatory system (15.9%), diseases of the digestive system (11.5%), and endocrine, nutritional and metabolic diseases (11.2%) (Table 1). Type of medical intervention is shown in Table 2. The proportion of main types was 49.9% for drug trials, 10.5% for physical therapy, and 8.5% for devices.

**Table 1:** Diseases and condition of all RCTs conducted in Japan (N = 1,013).

Diseases and conditions	Number	Percent
Diseases of the circulatory system	161	15.9%
Diseases of the digestive system	116	11.5%
Endocrine, nutritional and metabolic diseases	113	11.2%
Diseases of the musculoskeletal system and connective tissue	99	9.8%
Neoplasms	79	7.8%
Diseases of the respiratory system	66	6.5%
Mental and behavioral disorders	57	5.6%
External causes of morbidity and mortality	46	4.5%
Diseases of the nervous system	42	4.1%
Others	234	23.1%

**Table 2:** Type of intervention of all RCTs conducted in Japan (N = 1,013).

Type of intervention	Number	Percent
Drug	505	49.9%
Physical therapy	106	10.5%
Device	86	8.5%
Procedure/Surgery	78	7.7%
Biological/Vaccine	76	7.5%
Others	162	16.0%

Table 3 shows the design characteristics. The most frequent type of comparison group, study design and number of arms were the head-to-head trial (45.0%), parallel group design (75.2%) and two arms (77.1%), respectively. The median sample size was 42 (interquartile range: 23 to 101). While the number of subjects in 54.5% of RCTs was less than 50 (and notably, the proportion with sample size ≤20 was 17.3%), the sample size in 25.9% of studies was more than 100.

**Table 3:** Design characteristics of all RCTs conducted in Japan (N = 1,013).

Characteristics	Number	Percent
Type of comparison group		
Head to Head	456	45.0%
Placebo	394	38.9%
Dose	163	16.1%
Study design		
Parallel	762	75.2%
Crossover	205	20.2%
Factorial	46	4.5%
Number of arms		
2	781	77.1%
3	159	15.7%
≥4	73	7.2%
Sample size		
Less than 50	552	54.5%
50 to 99	199	19.6%
100 to 199	131	12.9%
200 to 499	87	8.6%
Over 500	44	4.3%

Of a total of 605 randomly-selected articles, only 131 articles (21.7%) reported their funding sources. The three most frequent reported funding sources were the Japanese government (41.2%), profit organizations (e.g. pharmaceutical companies) (38.9%), and foundations (9.2%). Other possible sources of funding were self-funding (0.7%), and financing from universities or research institutions (2.6%). Trials in which funding sources were unreported included drug (46.6%), physical therapy (10.8%) and device trials (10.3%). The proportion of description on trial registration was only 8.3%. The proportions of reported funding resources and trial registration among articles indexed in international databases were significantly higher than those non-indexed (p<0.001).

**Figure 2:**
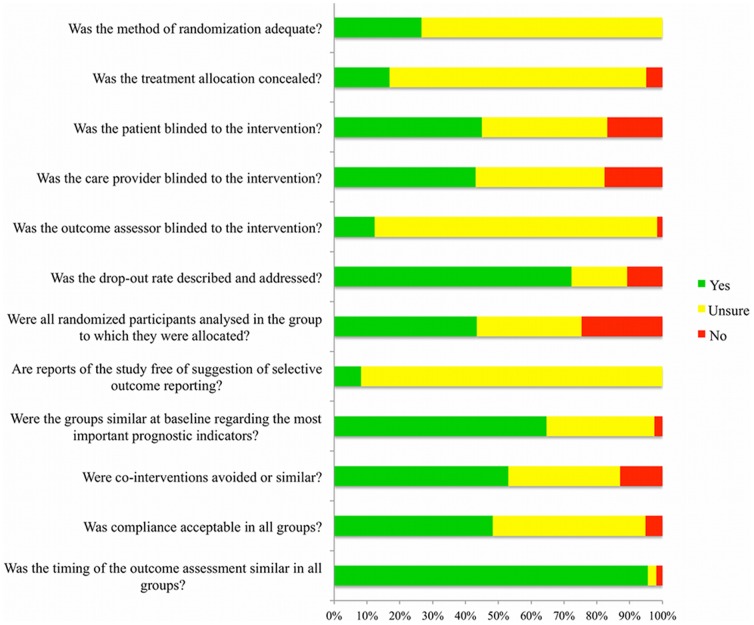
Quality assessment of Japanese RCTs.

The results of methodological quality of Japanese RCTs are presented in Figure 2. Of all 605 RCTs, 265 RCTs (43.8%) had more than six positive scores and without any fatal flaw (i.e., marked as “yes” for low risk of bias). The mean number of high quality item was 5.29 out of 12 domains. The proportion of “unclear” was relatively high and varied from 0% to 91.7%.

High risk of bias, where the proportion of “yes” was low (less than 33%), was observed in the following criteria: randomization (item 1), allocation concealment (item 2), assessor blinding (item 5) and selective outcome reporting (item 8). Medium risk of bias, where the proportion of “yes” was medium (33%–66%), was observed in the following criteria: patient blinding (item 3), provider's blinding (item 4), intention to treat (item 7), baseline similarity (item 9), co-intervention (item 10) and compliance (item 11). On the other hand, low risk of bias, where the proportion of “yes” was high (more than 66%), was observed in the following criteria: drop-out rate (item 6) and assessment timing (item 12).

Table 4 shows the comparison of quality of RCTs classified based on international indexing and publishers (explanations for classification of groups are listed in Table 4). The proportion of studies assessed as high quality that included the following criteria was significantly higher among Group A than Group C: method of randomization (item 1) (p = 0.02), allocation concealment (item 2) (p = 0.02), selective outcome reporting (item 8) (p<0.001) and similarity of baseline (item 9) (p = 0.004). The proportion of RCTs we assessed as high quality was 50.5% in Group A, 50.0% in Group B and 37.7% in Group C, respectively. Statistically significant difference was observed between Group A and C (p = 0.01).

**Table 4:** Comparison of quality of Japanese RCTs classified by international indexing and journal publisher's location.

	Group A	Group B	Group C
Total number	222	70	313
Proportion assessed as high quality	50.5%	50.0%	37.7%
1: Was the method of randomization adequate?	35.1%	20.0%	22.0%*
2: Was the treatment allocation concealed?	23.9%	10.0%*	13.4%*
3: Was the patient blinded to the intervention?	37.4%	51.4%	48.9%
4: Was the care provider blinded to the intervention?	40.5%	52.9%	42.8%
5: Was the outcome assessor blinded to the intervention?	15.8%	18.6%	8.6%
6: Was the drop-out rate described and addressed?	74.3%	72.9%	70.6%
7: Were all randomized participants analyzed in the group to which they were allocated?	50.0%	51.4%	37.1%
8: Are reports of the study free of suggestion of selective outcome reporting?	16.2%	7.1%	2.9%*
9: Were the groups similar at baseline regarding the most important prognostic indicators?	79.3%	77.1%	51.4%
10: Were co-interventions avoided or similar?	54.5%	47.1%	53.4%
11: Was the compliance acceptable in all groups?	41.4%	58.6%	50.8%
12: Was the timing of the outcome assessment similar in all groups?	96.8%	98.6%	93.9%

%: The proportion of “yes” in each criterion of the 2009 updated method guidelines for systematic reviews.

Group A: articles indexed in internationally databases and published in international journals.

Group B: articles indexed in internationally databases and published in Japanese journals.

Group C: articles non-indexed in international databases and published in Japanese journals.

*: p<0.05, compared with Group A by Holm's multiple comparison test.

Table 5 shows the result of regression analysis to determine the factors related to the high quality of RCTs. Surgical intervention studies for external causes of morbidity and mortality with a large sample size, trial registration and a large number of arms were most likely to be of a higher quality. As to type of disease and conditions, disease of the respiratory system and external causes of morbidity and mortality were higher quality than “others” category (p = 0.02, respectively), while as to type of intervention, device and procedure/surgery were associated with high quality (p = 0.001 and p<0.001, respectively) compared with a drug intervention. Larger sample size (100–200 participants) was correlated to quality (p = 0.004). Registration of the study protocol before trial implementation was positively correlated with quality (p = 0.003), as was number of arms (p = 0.03). However, quality was not related to international indexing in international databases and publishers compared to non-indexed Japanese databases (indexed Japanese journal: p = 0.07 and indexed foreign journals: p = 0.06).

**Table 5:** Factors related to quality of Japanese RCTs.

		Odds Ratio	95% CI	P-value
Disease and conditions				
	Others	Ref.	-	-
	Diseases of the circulatory system	1.07	0.59, 1.94	0.828
	Diseases of the digestive system	1.57	0.83, 2.96	0.161
	Endocrine, nutritional and metabolic diseases	1.34	0.70, 2.56	0.374
	Diseases of the musculoskeletal system and connective tissue	1.19	0.60, 2.36	0.615
	Neoplasms	0.90	0.41, 1.95	0.786
	Diseases of the respiratory system	2.48	1.16, 5.30	0.019
	Mental and behavioral disorders	1.14	0.49, 2.63	0.765
	External causes of morbidity and mortality	2.77	1.16, 6.61	0.022
	Diseases of the nervous system	2.54	0.98, 6.55	0.054
Type of intervention				
	Drug	Ref.	-	-
	Device	3.21	1.66, 6.24	0.001
	Biological/Vaccine	1.50	0.78, 2.90	0.221
	Procedure/Surgery	4.00	2.01, 7.96	<0.001
	Physical therapy	1.63	0.87, 3.04	0.126
	Others	1.42	0.83, 2.42	0.199
Comparison group				
	Head to head	Ref.	-	-
	Placebo	1.15	0.76, 1.74	0.500
	Dose	1.18	0.68, 2.04	0.561
Study design				
	Parallel	Ref.	-	-
	Cross	0.85	0.52, 1.38	0.500
	Factorial	0.67	0.25, 1.78	0.425
Sample size				
	<50	Ref.	-	-
	[50,100)	1.01	0.62, 1.63	0.974
	[100,200)	2.31	1.30, 4.09	0.004
	[200,500)	1.68	0.84, 3.36	0.140
	≥500	0.74	0.29, 1.88	0.527
Trial registration				
	No description	Ref.	-	-
	Description	3.01	1.45, 6.25	0.003
Number of arms		1.30	1.02, 1.66	0.034
International indexing and publishers				
	Non-indexed Japanese journal	Ref.	-	-
	Indexed Japanese journal	1.72	0.95, 3.11	0.074
	Indexed Foreign journal	1.51	0.98, 2.32	0.059
Description of funding				
	No description	Ref.	-	-
	Description	1.52	0.96, 2.4	0.072

CI: confidence interval.

## Discussion

This is the first study to assess the characteristics, quality and related factors of RCTs conducted in Japan. Despite a considerable number of RCTs conducted in Japan, their quality is not satisfactory in some domains. A total of 1013 RCTs conducted in Japan were published in 2010. Drug therapy was the most frequent type of intervention in Japan (49.9%). This result is similar to surveys among other countries, although the proportion of drug trials in Japan was higher than that in the UK, where 35% of all trials between 1980 and 2002 were drug trials [15]–[17]. Recently in Japan, clinical trial publications used by pharmaceutical companies as marketing tools for the drug Valsartan were withdrawn due to doubtful claims about the effectiveness of the drug [18], [19]. As a result, full disclosure and quality control of clinical trials has become a pressing research issue in Japan [20].

Although drugs have been central to many breakthroughs in healthcare, non-drug interventions such as early diagnosis and prevention, diets and nutrition also offer many benefits and tend to be cost-effective. According to the Global Burden of Diseases, Injuries, and Risk Factors Study 2010 in Japan, the top three causes of disability-adjusted life years (DALYs) in 2010 were lower back pain, cerebrovascular disease, and ischemic heart disease. The study strongly recommended the effectiveness of preventive interventions to improve DALYs [21]–[23]. Potential efficiency and efficacy of non-drug interventions are largely ignored in Japan. In the US, the Institute of Medicine Committee on Comparative Effectiveness Research Prioritization recommended research priorities by types of intervention, which put emphasis on systems of care, standards of care, behavioral treatment, prevention and procedures, as well as drugs [24], [25]. In addition, from the perspective of comparative effectiveness, head-to-head assessment, rather than comparison with placebo, is urgently needed to ensure effective decision making for both clinical practice and health policy [25]. However, according to this study, the proportion of head-to-head assessment in Japanese trials remained at 45%.

An interesting characteristic of Japanese RCTs is their sample size: more than half had a sample size less than 50 and the proportion with sample size ≤20 was over 17%. Although the sample size of trials should be decided based on both precision and statistical power, very small RCTs are often insufficient to evaluate benefits and risks of healthcare, their results have little generalizability [26], [27] and in large numbers they can introduce bias into meta-analysis [28]. Only 8.3% of Japanese trials were registered to a clinical trial registry system. In 2005, the International Committee of Medical Journal Editors required that all clinical trials be registered with a public registry before the enrolment of the first subject. The registry situation in Japan does not conform to this information disclosure rule, which is a key element in medical decision-making and preventing publication bias [29], [30]. Japan has three main registry systems, but there is no information on their coverage levels or on their relationship to other registry systems, and standardized key search words are not well developed. Also, it is difficult in some systems to search the registration date and more importantly the publication date of trials. Improvement of the Japanese registry system, and registry requirements for clinical trials, is urgently needed. In addition, approximately 80% of Japanese RCTs had no information on funding sources and around half of the studies that did not include funding information were drug trials. A systematic review of conflict of interests showed that systematic biases favored products made by the company funding the research [31]. Thus, the result in this study implies that there might be hidden conflicting interests and publication bias in Japan.

The present study showed that 44% of Japanese RCTs were high quality, as indicated by the 2009 updated method guidelines for systematic reviews [12]. Although RCTs not indexed in international databases were lower in quality than indexed RCTs in some items such as method of randomization, allocation concealment, selective outcome reporting and similarity of baseline, the overall quality was not significantly different between RCTs in indexed Japanese/foreign journals and non-indexed RCTs after adjusting for other factors in a logistic regression model. The non-indexed RCTs contained a considerable number of studies with high quality RCTs (37.7%). This result suggested that systematic reviewers should carefully examine non-indexed Japanese RCTs and should consider including both international and Japanese databases in their search strategies.

Another aspect of quality of RCTs was the reporting quality. In this study, a high proportion of “unsure” results were observed in each item of the 2009 updated method guidelines for systematic reviews. CONSORT statement was developed to respond to the demand for more sophisticated reporting quality of trials [32]. Several studies indicated that pre-CONSORT trials were inferior to post-CONSORT studies in several aspects of methodology and found that articles which adapted CONSORT statement showed the remarkable improvement in some specific reporting methods such as allocation concealment [6], [33], [34]. To improve the quality of Japanese RCTs, and increase the Japanese presence in the international academic community, the CONSORT statement should be encouraged for authors and publishers in Japan. Greater efforts are needed to improve the quality of Japanese RCTs as well as to include them in international databases and further research is needed to assess changes in quality longitudinally and conduct country by country comparisons.

## Supporting Information

Appendix S1 **Search Strategy.**
(DOCX)

Checklist S1 **PRISMA checklist.**
(PDF)
