# Supplementary material for: Are Japanese Randomized Controlled Trials Up to the Task? A Systematic Review
Source: PLoS One. 2014 Mar 3;9(3):e90127. doi: 10.1371/journal.pone.0090127 (PMC3940821; doi:10.1371/journal.pone.0090127)
Supplement: Appendix S1 — Search Strategy. (DOCX) [file pone.0090127.s001.docx]

Appendix S1: Search Strategy

| ★the Japan Medical Abstract Society Database | |
| --- | --- |
| 1 | ((ランダム化比較試験/RD) and (DT=2010)) and (CK=ヒト) |
|  |  |
| ★Ovid MEDLINE(R) | |
| 1 | Randomized Controlled Trial.pt. |
| 2 | (japan* or nihon or nippon or hokkaido or aomori or iwate or miyagi or akita or yamagata or fukushima or ibaraki or tochigi or gunma or gumma or saitama or chiba or tokyo or kanagawa or niigata or toyama or ishikawa or fukui or yamanashi or nagano or gifu or shizuoka or aichi or mie or shiga or kyoto or osaka or hyogo or hyougo or nara or wakayama or tottori or shimane or okayama or hiroshima or yamaguchi or tokushima or kagawa or ehime or kochi or fukuoka or saga or nagasaki or kumamoto or oita or miyazaki or kagoshima or okinawa or sapporo or sendai or yokohama or kawasaki or sagamihara or hamamatsu or nagoya or sakai or kobe or kitakyushu).in. |
|  |  |
| 3 | 1 and 2 |
| 4 | (retraction of publication or retracted publication).pt. |
| 5 | (japan* or nihon or nippon or hokkaido or aomori or iwate or miyagi or akita or yamagata or fukushima or ibaraki or tochigi or gunma or gumma or saitama or chiba or tokyo or kanagawa or niigata or toyama or ishikawa or fukui or yamanashi or nagano or gifu or shizuoka or aichi or mie or shiga or kyoto or osaka or hyogo or hyougo or nara or wakayama or tottori or shimane or okayama or hiroshima or yamaguchi or tokushima or kagawa or ehime or kochi or fukuoka or saga or nagasaki or kumamoto or oita or miyazaki or kagoshima or okinawa or sapporo or sendai or yokohama or kawasaki or sagamihara or hamamatsu or nagoya or sakai or kobe or kitakyushu).in. and random*.tw. |
|  |  |
| 6 | 4 and 5 |
| 7 | or /3,6 |
| 8 | Animals/ |
| 9 | Humans/ |
| 10 | 8 not (8 and 9) |
| 11 | 7 not 10 |
| 12 | limit 11 to yr="2010" |
|  |  |
| ★EMBASE.com | |
| #1 | randomized controlled trial'/de AND [embase]/lim NOT [medline]/lim |
| #2 | randomization'/de AND [embase]/lim NOT [medline]/lim |
| #3 | random*:ab,ti AND [embase]/lim NOT [medline]/lim |
| #4 | ('random effect' or 'random effects' or 'random regression' or 'random forest' or 'random forests' or 'random variable'):ab,ti AND [embase]/lim NOT [medline]/lim |
| #5 | #3 NOT #4 |
| #6 | rct*:ab,ti AND [embase]/lim NOT [medline]/lim |
| #7 | placebo'/de AND [embase]/lim NOT [medline]/lim |
| #8 | placebo*:ab,ti AND [embase]/lim NOT [medline]/lim |
| #9 | single blind procedure'/de AND [embase]/lim NOT [medline]/lim |
| #10 | double blind procedure'/de AND [embase]/lim NOT [medline]/lim |
| #11 | triple blind procedure'/de AND [embase]/lim NOT [medline]/lim |
| #12 | (singl* NEAR/1 (blind* OR mask*)):ab,ti AND [embase]/lim NOT [medline]/lim |
| #13 | (doubl* NEAR/1 (blind* OR mask*)):ab,ti AND [embase]/lim NOT [medline]/lim |
| #14 | ((tripl* OR trebl*) NEAR/1 (blind* OR mask*)):ab,ti AND [embase]/lim NOT [medline]/lim |
| #15 | retracted article'/de AND [embase]/lim NOT [medline]/lim |
| #16 | #1 OR #2 OR #3 OR #4 OR #5 OR #6 OR #7 OR #8 OR #9 OR #10 OR #11 OR #12 OR #13 OR #14 OR #15 |
| #17 | (book:it OR editorial:it OR letter:it OR review:it) AND [embase]/lim NOT [medline]/lim |
| #18 | nonhuman'/de AND [embase]/lim NOT [medline]/lim |
| #19 | #17 OR #18 |
| #20 | #16 NOT #19 |
| #21 | (japan* or nihon or nippon or hokkaido or aomori or iwate or miyagi or akita or yamagata or fukushima or ibaraki or tochigi or gunma or gumma or saitama or chiba or tokyo or kanagawa or niigata or toyama or ishikawa or fukui or yamanashi or nagano or gifu or shizuoka or aichi or mie or shiga or kyoto or osaka or hyogo or hyougo or nara or wakayama or tottori or shimane or okayama or hiroshima or yamaguchi or tokushima or kagawa or ehime or kochi or fukuoka or saga or nagasaki or kumamoto or oita or miyazaki or kagoshima or okinawa or sapporo or sendai or yokohama or kawasaki or sagamihara or hamamatsu or nagoya or sakai or kobe or kitakyushu):ad AND [embase]/lim NOT [medline]/lim |
| #22 | japan:ca AND [embase]/lim NOT [medline]/lim |
| #23 | #21 OR #22 |
| #24 | #20 AND #23 |
| #25 | #24 AND [2010]/py |
|  |  |
| ★CINAHL | |
| 1 | PT randomized controlled trial |
| 2 | MH "Randomized Controlled Trials" |
| 3 | MH "Random Assignment" |
| 4 | TI random* OR AB random* |
| 5 | TI rct* OR AB rct* |
| 6 | MH "Placebos" |
| 7 | TI placebo* OR AB placebo* |
| 8 | MH "Single-Blind Studies" |
| 9 | MH "Double-Blind Studies" |
| 10 | MH "Triple-Blind Studies" |
| 11 | TX ( (singl* n1 blind*) or (singl* n1 mask*) ) |
| 12 | TX ( (doubl* n1 blind*) or (doubl* n1 mask*) ) |
| 13 | TX ( (tripl* n1 blind*) or (tripl* n1 mask*) ) or TX ( (trebl* n1 blind*) or (trebl* n1 mask*) ) |
| 14 | MH "Retracted Publication" |
| 15 | S1 OR S2 OR S3 OR S4 OR S5 OR S6 OR S7 OR S8 OR S9 OR S10 OR S11 OR S12 OR S13 OR S14 |
| 16 | AF (japan* OR nihon OR nippon OR hokkaido OR aomori OR iwate OR miyagi OR akita OR yamagata OR fukushima OR ibaraki OR tochigi OR gunma OR gumma OR saitama OR chiba OR tokyo OR kanagawa OR niigata OR toyama OR ishikawa OR fukui OR yamanashi OR nagano OR gifu OR shizuoka OR aichi OR mie OR shiga OR kyoto OR osaka OR hyogo OR hyougo OR nara OR wakayama OR tottori OR shimane OR okayama OR hiroshima OR yamaguchi OR tokushima OR kagawa OR ehime OR kochi OR fukuoka OR saga OR nagasaki OR kumamoto OR oita OR miyazaki OR kagoshima OR okinawa OR sapporo OR sendai OR yokohama OR kawasaki OR sagamihara OR hamamatsu OR nagoya OR sakai OR kobe OR kitakyushu) |
| 17 | S15 AND S16 |
| 18 | S17 AND PY 2010 |
|  |  |
| ★PsycINFO(EBSCO) | |
| 1 | TI random* OR AB random* OR SU random* OR KW random* |
| 2 | TI rct* OR AB rct* |
| 3 | DE "Placebo" |
| 4 | TI placebo* OR AB placebo* OR SU placebo* OR KW placebo* |
| 5 | TX (singl* n1 blind*) or (singl* n1 mask*) |
| 6 | TX (doubl* n1 blind*) or (doubl* n1 mask*) |
| 7 | TX ( (tripl* n1 blind*) or (tripl* n1 mask*) ) or TX ( (trebl* n1 blind*) or (trebl* n1 mask*) ) |
| 8 | MR 2000 |
| 9 | S1 OR S2 OR S3 OR S4 OR S5 OR S6 OR S7 OR S8 |
| 10 | PO 10 |
| 11 | S9 and S10 |
| 12 | AF (japan* OR nihon OR nippon OR hokkaido OR aomori OR iwate OR miyagi OR akita OR yamagata OR fukushima OR ibaraki OR tochigi OR gunma OR gumma OR saitama OR chiba OR tokyo OR kanagawa OR niigata OR toyama OR ishikawa OR fukui OR yamanashi OR nagano OR gifu OR shizuoka OR aichi OR mie OR shiga OR kyoto OR osaka OR hyogo OR hyougo OR nara OR wakayama OR tottori OR shimane OR okayama OR hiroshima OR yamaguchi OR tokushima OR kagawa OR ehime OR kochi OR fukuoka OR saga OR nagasaki OR kumamoto OR oita OR miyazaki OR kagoshima OR okinawa OR sapporo OR sendai OR yokohama OR kawasaki OR sagamihara OR hamamatsu OR nagoya OR sakai OR kobe OR kitakyushu) |
| 13 | S11 and S12 |
| 14 | S13 AND PY 2010 |
